# *Helicobacter pylori*'s Unconventional Role in Health and Disease

**DOI:** 10.1371/journal.ppat.1000544

**Published:** 2009-10-26

**Authors:** Marion S. Dorer, Sarah Talarico, Nina R. Salama

**Affiliations:** Division of Human Biology, Fred Hutchinson Cancer Research Center, Seattle, Washington, United States of America; The Scripps Research Institute, United States of America

## Abstract

The discovery of a bacterium, *Helicobacter pylori*, that is resident in the human stomach and causes chronic disease (peptic ulcer and gastric cancer) was radical on many levels. Whereas the mouth and the colon were both known to host a large number of microorganisms, collectively referred to as the microbiome, the stomach was thought to be a virtual Sahara desert for microbes because of its high acidity. We now know that *H. pylori* is one of many species of bacteria that live in the stomach, although *H. pylori* seems to dominate this community. *H. pylori* does not behave as a classical bacterial pathogen: disease is not solely mediated by production of toxins, although certain *H. pylori* genes, including those that encode exotoxins, increase the risk of disease development. Instead, disease seems to result from a complex interaction between the bacterium, the host, and the environment. Furthermore, *H. pylori* was the first bacterium observed to behave as a carcinogen. The innate and adaptive immune defenses of the host, combined with factors in the environment of the stomach, apparently drive a continuously high rate of genomic variation in *H. pylori*. Studies of this genetic diversity in strains isolated from various locations across the globe show that *H. pylori* has coevolved with humans throughout our history. This long association has given rise not only to disease, but also to possible protective effects, particularly with respect to diseases of the esophagus. Given this complex relationship with human health, eradication of *H. pylori* in nonsymptomatic individuals may not be the best course of action. The story of *H. pylori* teaches us to look more deeply at our resident microbiome and the complexity of its interactions, both in this complex population and within our own tissues, to gain a better understanding of health and disease.

Common wisdom circa 1980 suggested that the stomach, with its low pH, was a sterile environment. Then, endoscopy of the stomach became common and, in 1984, pathologist Robin Warren and gastroenterologist Barry Marshall saw an extracellular, curved bacillus, often in dense sheets, lining the stomach epithelium of patients with gastritis (inflammation of the stomach) and ulcer disease [1]. Soon, the medical community understood that the gram-negative bacterium *Helicobacter pylori*, not stress, is the major cause of stomach inflammation, which, in some infected individuals, precedes peptic ulcer disease (10%–20%), distal gastric adenocarcinoma (1%–2%), and gastric mucosal-associated lymphoid tissue (MALT) lymphoma (<1%) [2]–[5]. Thus, *H. pylori* gained distinction as the only known bacterial carcinogen [6]. It is believed that half of the world's population is infected with *H. pylori*; however, the burden of disease falls disproportionately on less-developed countries. The incidence of infection in developed countries has fallen dramatically, for unknown reasons, with a corresponding decrease in gastric cancer [7]. This public health success is tempered by the recent demonstration of an inverse relationship between *H. pylori* infection and esophageal adenocarcinoma, Barrett's esophagus, and reflux esophagitis [8]. *H. pylori* has been with humans since our earliest days, thus it is not surprising that its relationship is that of both a commensal bacterium and a pathogen, causing some diseases and possibly protecting against others. In addition, it is genetically diverse, likely as a result of constant exposure to both environmental and immunological selection, suggesting that genetic diversification is a strategy for long-term colonization.

## The Role of Infection in Disease Risk

**Figure 1:**
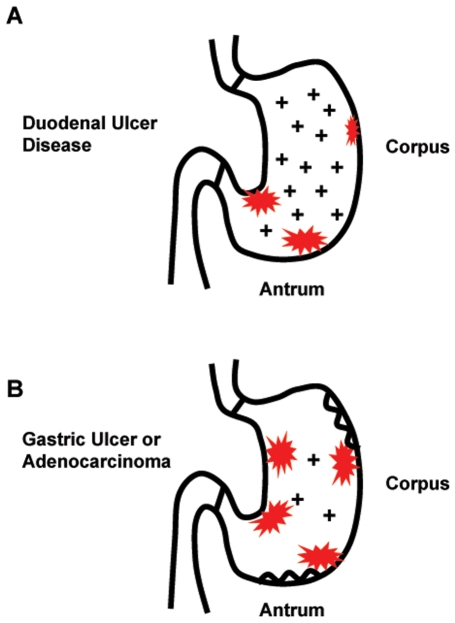
Distinct pathologies of *H. pylori*–induced disease. (A) Duodenal ulcer disease correlates with high inflammation in the antrum (red bursts), lower levels of inflammation in the corpus, and high acid secretion (+). (B) Gastric ulcer or adenocarcinoma correlates with increased inflammation in the corpus, low acid secretion, and multifocal atrophy (wavy lines).

*H. pylori* infection is generally acquired during childhood and, without specific antibiotic treatment, can persist for the lifetime of the host. Disease often does not develop until adulthood, after decades of infection, and *H. pylori* induces variable pathologies in the stomach. Duodenal ulcer disease is characterized by gastritis that is largely confined to the antrum (the distal compartment of the stomach), relatively low inflammation of the corpus (the middle, acid-secreting compartment), and high levels of stomach acid secretion (Figure 1A). Those with gastric ulcer or stomach cancer have high levels of inflammation of the corpus, multifocal gastric atrophy, and low levels of stomach acid secretion, due to the destruction of stomach acid–secreting parietal cells (Figure 1B) [9],[10]. Some of this inflammatory response is controlled by the cytokine IL-1β, which is induced by *H. pylori* infection [11] and both elicits a proinflammatory response and inhibits secretion of gastric acid [12]. Polymorphisms in the interleukin gene cluster, including *IL-1β*, are risk factors for *H. pylori*–associated gastric cancer [13],[14], and studies of the transcriptional response of both human and model hosts to *H. pylori* confirm induction of transcriptional regulators of proinflammatory programs. In addition, transcription profiles reveal induction of several chemokines and cytokines including those produced by nonlymphoid cells, and robust induction of innate immune defenses including iron sequestration proteins and antimicrobial peptides [15]. These studies suggest it would be wise to explore diverse functional classes of genes for host genetic variant associations with *H. pylori* disease progression. To this end, *H. pylori* researchers are eagerly awaiting an unbiased genome-wide association study of risk factors associated with progression to intestinal-type gastric cancer or peptic ulcer disease in patients infected with *H. pylori*. Such a study has been completed for sporadic diffuse-type gastric cancer, which can be associated with *H. pylori* infection, revealing two candidate loci, one that encodes a likely tumor suppressor (prostate stem cell antigen [PSCA]) [16]. Genomic studies of this sort will help elucidate host factors that synergize with *H. pylori* infection to cause disease.

The association of *H. pylori* infection with gastric cancer raises the interesting question of whether *H. pylori* encodes one or more oncogenes. Oncogenic viruses initiate and promote cellular transformation by integrating virally encoded oncogenes into the host genome [17],[18]. By contrast, *H. pylori* remains primarily extracellular and does not integrate its genome into the host DNA. The bacterium can still affect the function of host cells, however, by translocating a bacterial protein, CagA, into host cells via a specialized secretion system called the *cag* Type IV secretion system (T4SS) [19],[20]. In host cells, CagA interacts with a number of cellular complexes implicated in oncogenesis [21],[22]. Despite elucidation of potentially transforming activities, transgenic expression of CagA in the mouse stomach is only weakly oncogenic [23]. As the *cag* T4SS also induces proinflammatory cytokines via the intracellular bacterial peptidoglycan recognition molecule Nod1, cancer progression may occur through synergy with the host inflammatory response [24]. While CagA may not promote cancer itself, exposure to CagA and inflammatory insults may select for heritable host cell changes (genetic or epigenetic) that together contribute to cancer progression.

*H. pylori* expands our view of how microbes survive at high levels while activating inflammatory responses and shows us that microbes may be underappreciated as an important factor in chronic disease pathogenesis. In the case of pathogens that cause acute infections, there is a massive inflammatory response, which often supports bacterial replication and transmission. Alternatively, some pathogens, such as *Mycobacterium tuberculosis*, persist in the host by manipulating the immune response to create a protected compartment. *H. pylori* introduces a third strategy; it actively replicates and maintains a continuous balance with the inflammatory response over years of infection with little evidence for increased *H. pylori*–related disease upon immune suppression [25]. As the role of chronic inflammation in many diseases including cardiovascular disease, diabetes mellitus, Alzheimer's disease, and others is increasingly recognized, researchers are focusing on infectious agents as one possible source of this chronic inflammation.

## Genomic Insights into the Biology of *H. pylori*

The study of *H. pylori* is strongly influenced by the genomic age. The sequencing of its genome was completed in 1997 [26], just 13 years after Marshall and Warren reported their discovery. However, almost a quarter (24%) of *H. pylori* genes have no sequence similarity with genes available in public databases [27], suggesting that lessons learned from well-studied bacteria like *Escherichia coli* would not necessarily apply to this evolutionarily distinct Epsilonproteobacteria. By using more advanced bioinformatic approaches, researchers are now identifying some pathways first thought absent in *H. pylori*. For example, *H. pylori* appeared to lack the *E. coli recBCD* pathway, which is involved in homologous recombination and DNA double-strand break repair. More careful examination of conserved domains and motifs, however, identified the *H. pylori addA* and *addB* genes, which are present in most gram-positive and many gram-negative bacteria and whose protein products have enzymatic functions similar to those of the *recBCD* pathway [28].

By 1999, *H. pylori* was the first species to have complete genomes sequenced from two different strains—an important milestone, given its genetic diversity. Comparison of the two genomes revealed that 6%–7% of the genes were present in one strain but not in the other. There was also a high level of nucleotide diversity between the two strains, with only eight genes sharing at least 98% nucleotide identity; however, most nucleotide differences were synonymous changes [27]. Microarrays designed upon these sequences were then used for comparative genomic hybridization of *H. pylori* strains isolated from different ethnic groups and geographic areas [29],[30]. These studies found that 25% of *H. pylori* genes are variably present among strains. Such genome-wide analyses have played an important role in dividing *H. pylori* genes into two classes: variable genes that are absent in some strains and core genes that are present in all strains analyzed. The variable genes are likely adaptive for different environmental niches, which for the human stomach–restricted *H. pylori* comprise genetically distinct hosts. The largest annotated class of variable genes encode proteins expressed on or that modify the bacterial cell surface (outer membrane proteins and proteins involved in lipopolysaccharide synthesis) [30], consistent with a function at the interface of the bacteria and host. The core genes have diverse functions. Some core genes are required for viability in culture. A genomic study that utilized microarray-based mapping of a genome-saturating transposon library (a collection of *H. pylori* strains that includes transposon mutants randomly distributed throughout the genome) revealed that 23% of the genome is required for viability in culture because these genes could not tolerate transposon insertion [31]. Additional core genes are essential only in the context of host infection and several groups have completed screens for transposon mutants that fail to colonize animal models of infection [32],[33]. An example of such a colonization core gene is *addA*, which is required for recombinational repair of DNA double-strand breaks, presumably caused by the host inflammatory response [28].

The nucleotide sequence diversity in *H. pylori*'s core genes can distinguish between different ethnic and geographic human populations, demonstrating that passage of *H. pylori* between closely related humans has continued uninterrupted over tens of thousands of years (see Box 1). Different geographic and ethnic groups that have similar infection rates have quite varied relative risks of *H. pylori*–associated diseases such as gastric cancer [34]. Thus, in addition to host genetic and environmental exposures, differences among strains likely contribute to variation in disease risk. Consequently, studies of pathogenesis need to be reproduced in representative strain backgrounds to ensure that discoveries in one strain apply in strain populations with a diverse evolutionary history.

**Box 1. Tracking Human Genealogy with *H. pylori* Genomics**

Currently, a number of companies propose to predict your “genetic genealogy” from the DNA in a cheek swab. They do this by analyzing informatively variable parts of our genomes (such as the Y chromosome or mitochondrial DNA) that show characteristic differences between ethnic and geographic populations; thus, they can tell if you may be distantly related to Ghengis Khan, for example. Unfortunately, population bottlenecks [51], small population sizes, and long generation times have limited the amount of genetic diversity in the human population that can be used for these analyses. It turns out, however, that genomic sequencing of the *H. pylori* strain harbored by an individual does a better job in resolving ancestry than the usual human genomic markers [52]. This is because of high genetic diversity among *H. pylori* strains [53], a restricted mode of transmission (primarily within families or households [54]), and the association of *H. pylori* with humans throughout our evolution [55]. A major source of *H. pylori*'s genetic diversity is recombination between strains [38], which blurs signatures of descent. Despite this confounding factor, Achtman and colleagues [53] identified evolutionary signatures in strain sequences from diverse geographic sources. These signatures, combined with new statistical tools that take into account admixture and recombination [55], have tracked ancient human migrations, such as our emergence from Africa [55], and more recent events such as colonization of the Pacific islands [56]. *H. pylori* gene sequences can even distinguish between the Buddhist and Muslim ethnic groups that have coexisted for at least 1,000 years in Ladakh [52]. The fact that *H. pylori* has maintained evolutionarily distinct strain signatures during many generations of contact suggests either that interracial interactions that promote transmission are very limited or that additional mechanisms prevent strains from one ethnic population from establishing a foothold in hosts of another ethnic population.

## *H. pylori* Diversification during Persistent Infection

Genetic diversification can aid in the persistence of organisms that continue to replicate during chronic infection, allowing them to sample adaptive variants. HIV, for example, has a flexible reverse transcriptase that makes point mutations, insertions, deletions, transversions, and duplications that produce variants that may have a selective advantage [35]. Genetic variation in a microbe indicates constant selection by a dynamic environment, and *H. pylori* is a very genetically diverse species of bacteria [36]–[38]. Genetic diversification may help *H. pylori* to adapt to a new host after transmission, to different micro-niches within a single host, and to changing conditions in the host over time—for example, by avoiding clearance by host defenses.

**Figure 2:**
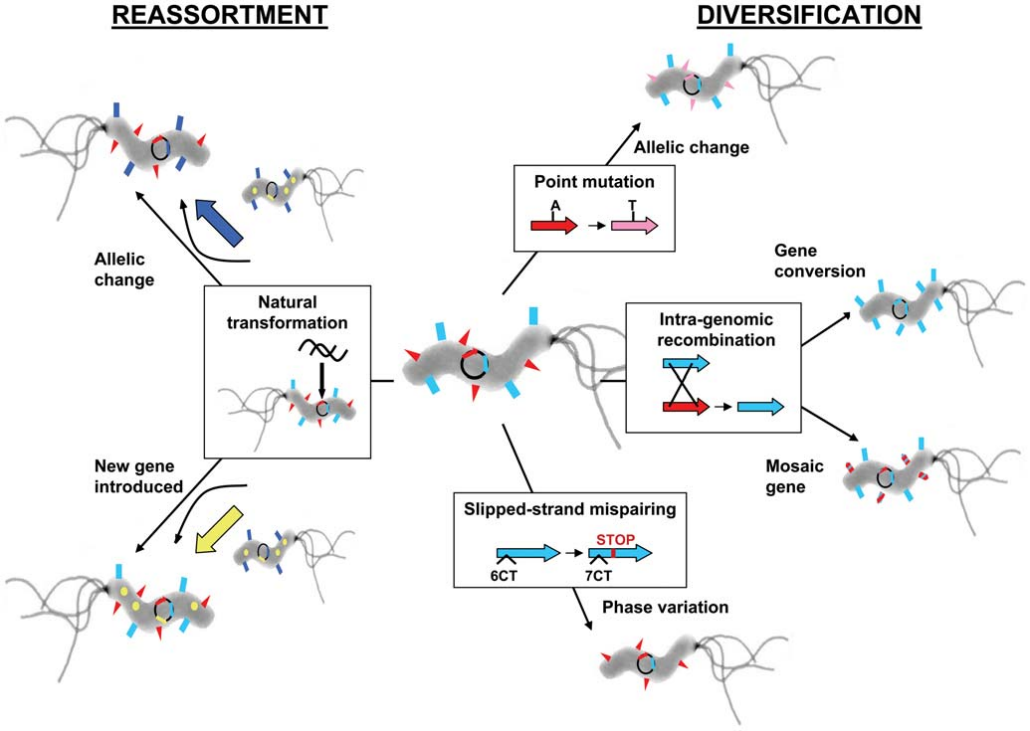
Mechanisms that create genetic diversity in *H. pylori*. Colored arrows represent different genes, and the correspondingly colored triangles, rectangles, and circles represent the proteins encoded by these genes. Diversification mechanisms (right side of figure) include spontaneous point mutations, slipped-strand mispairing, and intragenomic recombination. Allelic changes involving nonsynonymous point mutations and mosaic genes resulting from intragenomic recombination can alter the function and/or the antigenic epitopes of the encoded protein. Gene expression can also be regulated by gene conversion resulting from intragenomic recombination, and phase variation mediated by slipped-strand mispairing. Reassortment of genes (left side of figure) by natural transformation with exogenous DNA also contributes to genetic diversity. Natural transformation with DNA from a superinfecting strain, for example, can introduce new genes and new alleles of already present genes (horizontal gene transfer). Similarly, natural transformation with DNA from a variant clone of the same strain can further propagate an advantageous allele acquired by within-genome diversification.

Genetic diversity arises from within-genome diversification as well as from reassortment by recombination with DNA from other infecting *H. pylori*, generating novel clones within the stomach (Figure 2). Within-genome diversification can include point mutations, intragenomic recombination, and slipped-strand mispairing during DNA replication within repetitive sequences. Reassortment can occur by recombination with either DNA from a superinfecting *H. pylori* strain or a variant clone of the same strain. Central to this reassortment is *H. pylori*'s natural competence—the ability to take up exogenous DNA and incorporate it into its genome. Evidence from our lab shows that natural competence is induced by DNA damage, suggesting that *H. pylori* responds to stress by diversifying its genome (MSD and NRS, unpublished data). However, there are controls on this rampant genetic exchange: restriction-modification systems, which include a restriction endonuclease that cleaves a specific DNA sequence and a DNA methyltransferase that protects the bacterium's own DNA from being cleaved by methylating the target DNA sequence. Genes that encode restriction-modification systems compose the second largest class of variably present genes with known function, so the complement of available restriction-modification systems varies between strains, giving a methylation code to the DNA from each strain. This mechanism serves to limit or prevent recombination between *H. pylori* strains as well as between *H. pylori* and other bacteria or eukaryotic cells [39].

The *H. pylori* genome encodes relatively few proteins that regulate transcription. Instead, some of the same processes that govern the generation of genetic diversity (i.e., slipped-strand mispairing, methyltransferase activity, and recombination) also play an important role in varying gene expression in response to environmental cues. There are 46 *H. pylori* genes that have long repeats of one or two nucleotides that are prone to slipped-strand mispairing during replication [26],[27],[40]. These genes are phase-variable because changes in the number of repeats can shift the reading frame of the gene, switching gene expression on or off (Figure 2). In addition, many *H. pylori* promoters have mononucleotide repeats that regulate gene expression by changing the spacing between important regulatory sites in these promoters. Orphan methyltransferases, which have lost their corresponding restriction enzyme, may also regulate gene expression by methylating sequences in the promoter region of genes, and some of the methyltransferase genes are themselves subject to phase-variable expression. Recombination regulates gene expression through deletions and duplications that occur during gene conversion and locus switching. These mechanisms suggest that *H. pylori* survives by constantly generating variants that adapt its physiology to new environments.

One example of how *H. pylori*'s genetic variability helps it adapt to new environments involves its adhesin genes, which encode proteins that bind to the Lewis human blood group antigens, which are carbohydrate-based epitopes [41]. The protein encoded by one of these adhesin genes, BabA, binds the Lewis-b antigen on the gastric mucosa, helping the bacterium adhere to the mucosa. The *babA* gene is silent in some *H. pylori* strains but can be expressed if it recombines with the *babB* gene, an event mediated by homologous sequences at the 5′ and 3′ ends of the two genes [42]. Thus, recombination can help *H. pylori* alter its adherence properties to adapt to selective pressures in the host. These selective pressures may include variation in the host receptors present or in conditions that favor a shift in the ratio of bacteria adherent to the gastric cell epithelium over those swimming freely in the mucus.

Genetic variation may also be important for the ability of *H. pylori* to evade the host immune system. *H. pylori* further exploits the Lewis antigen system by “camouflaging” its surface lipopolysaccharide with its own Lewis-type antigen, which mimics that of the individual host. The bacterium adapts the spectrum of Lewis antigens it expresses by phase variation of the genes involved in their biosynthesis [43]. Furthermore, recombination among the many members of the large outer membrane protein (*omp*) gene family has the potential to create mosaic *omp* genes, generating antigenic variation that may keep *H. pylori* ahead of the ability of the host's immune system to recognize these cell surface exposed epitopes.

## *H. pylori*'s Interaction with the Microbiome

*H. pylori* share their niche with the stomach microbiome, the collection of microorganisms living on and in us. Study of microorganisms was once limited to only those microbes that could be cultured in the laboratory. Advances in sequencing technology now allow us to study the collection of genes encoded by any group of organisms—so-called metagenomics—making it possible to characterize also the microbes that cannot be cultured but nevertheless affect our health. Given that *H. pylori* engages in DNA exchange, the metagenome may serve as a repository for novel traits. When present, *H. pylori* dominates the microbiome in the stomach [44],[45], although the effect of this dominance is not known. Perhaps *H. pylori* infection changes the composition of the stomach microbiome, with unknown consequences.

## Challenges for the Future

*H. pylori* is considered pathogenic, even carcinogenic. With this simple view, eradication seems an obvious choice. In reality, however, the relationship between *H. pylori* and disease is more nuanced. Like the cancer risk associated with smoking, a recent trial showed that the cancer risk from *H. pylori* diminished measurably only 12 years after eradication of the infection [46]. Some studies suggest that infection may prevent diseases of the esophagus, and there is a debate in the literature concerning a relationship between *H. pylori* and childhood asthma [8],[47],[48]. There is clear consensus that *H. pylori* should be eliminated in cases of peptic ulcer disease, gastric MALT lymphoma, early gastric cancer, first-degree relatives of gastric cancer patients, and uninvestigated dyspepsia in high-prevalence populations. Despite its potential to prevent ulcer and cancer, universal eradication of *H. pylori* infection has not gained wide support, because of the mixture of positive and negative disease associations with infection, the lack of a definitive bacterial or host molecule accounting for disease causation, and poor success rates of treating non-ulcer dyspepsia by clearing *H. pylori* infection [49],[50]. Thus a more detailed picture of this host–pathogen interaction is needed and likely will depend upon further advances in both endoscopy and genomics.

We have a poor understanding of the immune responses to *H. pylori* and the reasons that most hosts fail to clear infection. The host restriction of *H. pylori* to humans and some nonhuman primates has hampered development of robust animal models to study the disease process. Thus progress will require improvements in animal models and improved access to patient samples. Endoscopy of the upper gastrointestinal tract is an invasive procedure, so a major limitation to research is collection of bacterial and human tissue samples from infected people. Available samples are biased toward patients with severe dyspepsia, ulcer symptoms, and gastric cancer, and only a small fraction of the stomach can be sampled. Advances in less-invasive methods, such as capsule endoscopy, may allow increased sampling to monitor bacterial and tissue changes during chronic colonization, including isolation and phenotypic analysis of immune effector cells in infected tissue. Less-invasive methods would also provide an opportunity to study infection in asymptomatic individuals and transmission of *H. pylori* infection, conditions in which the selective pressures that drive the observed *H. pylori* genetic diversification likely operate.

A major opportunity to increase our understanding of how *H. pylori* causes or prevents disease arises from recent advances in high-throughput sequencing technologies. Currently, several platforms allow researchers to accomplish in a single experiment sequencing or resequencing of tens of *H. pylori* genomes, characterization of host immune and epithelial cell types that change during infection with highly sensitive digital expression tag analysis, or analysis of the microbiome present in the stomach and esophagus through metagenomic sequencing or targeted bacterial or fungal small ribosomal subunit DNA sequencing. The sequence data generated by such experiments will address several important mysteries of *H. pylori* biology, including the timing and extent of *H. pylori* genetic diversification. While strains from unrelated individuals show dramatic variation in gene content and gene sequence, the extent of sequence variation among clones during persistent infection of a single host or upon transmission has not been adequately sampled. Whole-genome sequencing of multiple isolates of individual patients with dense spatial and temporal sampling would definitively establish when, where, and by what mechanisms genetic diversity is generated. This information will inform efforts to combat resistance to current antibiotics, to develop vaccines, and to understand *H. pylori*'s coevolution with humans. Exploration of the influence of *H. pylori* on the microbiome will identify organisms that collaborate with or can be antagonized by *H. pylori*. Such organisms may mediate some of the disease risks that have been associated with *H. pylori* presence and absence. Finally, the rapid pace of resequencing of *H. pylori*'s human host will provide a deeper understanding of genetic variation in the human population that may influence risk for *H. pylori*–associated pathologies and which, by association, could provide clues to the cellular pathways disrupted in disease. Thus, genomic approaches to study host response, the human microbiome, bacterial genetic variation, and, perhaps most importantly, the intersections among these components, will help researchers determine whether eradication is appropriate for all individuals in all populations.
